# Knowledge, Attitudes, Behavior, and Practices of Self Breast Examination in Nicaragua

**DOI:** 10.7759/cureus.21317

**Published:** 2022-01-17

**Authors:** Gabrielle Franco, Igor Martin R Herrera, Karen Vanessa H Castro, Vijay K Chattu, Thankam Sunil

**Affiliations:** 1 Emergency and Acute Infectious Disease Unit, Texas Department of State Health Services, Austin, USA; 2 Public Health, University of Guadalajara, Guadalajara, MEX; 3 Evaluation, Hospital Militar Escuela Dr. Alejandro Dávila Bolaños, Managua, NIC; 4 Medicine, University of Toronto, Toronto, CAN; 5 Public Health, University of Tennessee, Knoxville, USA

**Keywords:** behavior, attitude, knowledge, rural, urban, nicaragua, self-breast examination

## Abstract

Self breast examination (SBE) has been recommended as an important preventative practice for the early identification of breast cancer in women. However, our understanding of women’s knowledge, attitudes, and practices of self breast examination in Nicaragua is limited. In the present study, we conducted a cross-sectional study of women aged 18 years and over (n=500) living in selected urban and rural areas of Nicaragua. Measures included the survey reflected knowledge, attitudes, behaviors, and practices related to self breast examination. We compared these measures between women living in urban and rural areas and women aged 18-39 years and 40+ years. Using a t-test, we tested the significance of these differentials. Results indicate widespread and significant differentials in basic knowledge and behaviors on self-breast examination practices among women living in rural and urban locations in Nicaragua. Further, while younger women reported significant and lower overall knowledge about breast cancer (BC), purpose and reasons for SBE, characteristics of women who tend to be at higher risk for BC, and strategies and steps women take performing SBE compared to women who were in the 40+ age group. Study results call for location and population-specific programs and policies addressing disparities in breast cancer prevention efforts in the country.

## Introduction

According to the latest figures, around 508,000 women die annually from breast cancer [1], and it was highlighted as a great threat to women's health around the world [2]. In 2018, breast cancer deaths stood at 627,000, representing nearly 15% of all recorded cancer deaths among women worldwide [3], indicating an upsurge of approximately 119,000 breast cancer deaths from 2017. According to the global burden of diseases study 2017, the levels and trends in causes of disability-adjusted life years (DALYs) of breast cancer and risk factors showed similarities between 2007 and 2017. However, there are some differences that can be attributed to high fasting plasma glucose and high body mass index in countries across the world. [4]. Although developed countries suffer more from the recorded cases of the disease, in 2011, 50% of breast cancer death cases were from 58% of developing countries [5]. As mentioned, breast cancer has quickly become one of the leading causes of death among women worldwide; even though it can be detected easily through proper healthcare access and screening methods, its prevalence continues to rise. This is due to a severe lack of essential healthcare measures and services in low-income communities around the world. Quality of care and prevention is usually dependent on the systems put in place, such as effective screening programs and proper resources [6]. In countries that have limited resources, this can prove to be a difficult measure to accomplish. Nicaragua, a country that continues to struggle with efficiency in health care facilities and resources, has found access to medicines and certain medical tests difficult for its citizens, especially the poor [7]. This lack of access contributes to the inability to practice prevention and screening methods against communicable and non-communicable diseases. Breast cancer, which is considered a non-communicable disease, has become highly prevalent in recent years, affecting females in the Central and South American (CSA) regions with 140,000 new breast cancer diagnoses and 40,000 cancer deaths. It is estimated that by 2030, female breast cancer will increase in CSA regions by 70% (224,000 new cases and 66,000 cancer deaths) [8].

There are a wide variety of factors that can contribute to why breast cancer is one of the leading causes of death among women. Environmental, social, economic, and demographic factors are just a few examples contributing to breast cancer. Data has found that middle and low-income regions bear a disproportionate burden of breast cancer-related mortality [9]. For instance, in 2009, Nicaragua's death rate from female breast cancer was almost half as high as the death rate from cervical cancer - 11.1 and 19.4 deaths per 100,000 females, respectively [10]. Other factors, such as knowledge, beliefs, and perceived risk of breast cancer, can also contribute to low screening and preventative adherence. Latinas are most likely to be diagnosed with advanced breast cancer (stages IIb or higher on histopathology results), and this rate can account for the suboptimal rates of mammography adherence in the Latina population [11]. 

Self breast examinations (SBE) are effective in the early detection of lumps or cysts, which could indicate breast cancer [12]. Not only are SBEs useful in early detection, but they are cost-effective because women can perform these exams on their own at home. By performing SBE monthly, the chance of early detection is increased, and adherence to clinical examinations is improved [13]. However, to ensure that the practice of the SBE probe is effective, it is crucial to provide the necessary knowledge on how to perform them. A recent study conducted in Nicaragua showed that most women (91.9%) never performed a clinical examination performed, nor had they ever performed an SBE [14]. One barrier that researchers and many Latina women face regarding SBE is the cultural barriers associated with healthcare and preventative practices for illnesses such as breast cancer. Many Latin culture women hold family and traditions to a high standard that typically take precedence over their health. Fear, stigma, influences from social circles, and taboo associated with breast cancer increase Latina women's possibility to hold back from taking necessary preventative steps such as SBE and clinical examination [15].

It has also been found that middle-aged adult women's health concerns have little to no attention in low resource settings such as Nicaragua [16]. Therefore, it is important to continue to explore the vast subject of breast cancer, breast cancer prevention, and its effects on the Latino population. The purpose of this study is to measure and explore knowledge, attitudes, beliefs, and practices (KABP) about breast cancer and practices of SBE through a survey applied to women aged 18 years and older living in the urban and rural areas of Nicaragua.

## Materials and methods

Data collection for the study was conducted during the months of March to April 2019. We conducted a cross-sectional survey measuring different measures of KABP among women aged 18 years and over who were living in selected urban and rural areas of Nicaragua. For the urban location, women living in Managua municipality, and for the rural location, women living in San Marcos and Ometepe municipalities were selected. These two locations significantly differ in terms of social and economic characteristics of the population residing at these locations and of access to services. These two locations significantly contrast in terms of the differentials in basic services available in the country's urban and rural areas{17}. In general, in 2014, 60 percent of the rural and 36.5 percent of the urban population were living in poverty, and also in 2015, approximately 56 percent in the rural population were using improved sanitation facilities; this percentage was over 76 percent for a population living in urban areas [17]. We interviewed 250 women from each location to a total of 500 women for the present study. Using G*Power, for a sample of 500 would have an effect size of 0.3, with power 0.9 and alpha=0.05. The survey included a total of 132 questions grouped into eight indexes, aimed at capturing women's KABP. The survey questions included in our study were based on indexes that had been validated in other studies [18,19,20]. To carry out the surveys at the selected municipalities, we recruited a group of nursing students from a local nursing school who know the local communities, provided them with adequate training in conducting interviews, recruitment strategies, and protocols before implementing the survey. 

Inclusion criteria were women a) aged 18+ years and older, b) lived at the selected municipalities, and c) consented to voluntary participation. However, women survivors of other forms of cancer were excluded from the study. Nursing students approached women at public spaces in each municipality, central squares, and at streets adjacent to the plaza throughout the day. In total, the sample size of 500 women was selected, considering the criteria mentioned above to meet the research objectives. 

Based on the 132 items of the survey, eight indexes were created to compare KABP about breast cancer and SBE according to the location of residence in either urban or rural areas: knowledge about breast cancer (KBC), knowledge about breast cancer screening (KCS), frequency and timing of self breast examination (F&T), purpose or reasons for self-breast examination (PoRs), characteristics of women recommended highly for self breast examination attitudes (CoW), strategies or steps recommended for self breast examination (SoS), barriers to examination (BtE) and attitudes toward self breast examination (ASBE). Each index was calculated on an additive scale, and a higher value represents a higher representation of the character that represents the index. For example, a higher value in the BtE index shows more barriers, and a higher CoW index reflects a better understanding of the characteristics of respondents who are more likely to be performing a self breast examination and so on. The indicators included knowledge about breast cancer and screening, timelines of examinations, purpose, strategies, barriers, and attitudes towards the self breast examination. Then, descriptive statistical analysis was performed to obtain frequencies and percentages concerning the characteristics of the participants. Using a t-test, we compared the eight indexes between two stratified groups, namely area of residence (urban or rural) and age group (18-39 or 40+). 

The study was evaluated and approved by the research ethics committee at the Faculty of Medicine of the Army in Nicaragua in collaboration with the University of Texas at San Antonio.

## Results

Table 1 provides the background characteristics of all respondents included in the study. These characteristics include age, location of residence, educational level, occupation and marital status of respondents.

**Table 1:** Profile of all respondents

Characteristics	N	%
Age		
18-39 yrs.	285	57
40+ yrs.	215	43
Location		
Rural	250	50
Urban	250	50
Education		
Illiterate	26	5.2
Primary	156	31.2
Secondary	141	28.2
Upper middle	31	6.2
Bachelor	136	27.2
Post-graduate	10	2
Occupation		
Housewife	278	55.6
Student	36	7.2
Dependent worker	126	25.2
Freelance worker	60	12
Marital status		
Single	181	36.2
Married	194	38.8
Free union	100	20
Divorced	13	2.6
Widow	11	2.2

It was observed that 57 percent of the respondents were in the age group 18-39 years. Less than one-third (31.2 percent) of the respondents had only a primary education level, followed by 28.2 percent with secondary level education and 27.2 percent with bachelor-level education. A majority of the respondents (55.6 percent) were engaged. One-quarter of the respondents (25.2 percent) were dependent workers; approximately 39 percent were married, 6.2 percent were single, and 20 percent of the respondents were living in free unions. 

 Table 2 provides a description of all the measures included in the study about KABP indexes. 

**Table 2:** Overall mean, standard deviation, and range for indicators SBE - self breast examination

	N	Mean	Standard deviation	Min-max
Knowledge about breast cancer (KBC)	499	14.32	2.11	7.00-19.00
Knowledge about breast cancer screening (KCS)	500	2.91	0.74	.00-5.00
Frequency and timing (F&T)	499	22.15	4.69	16.00-41.00
Purpose or reasons (PoRs)	500	13.08	1.54	4.00-14.00
Characteristics of women (CoW)	500	6.96	1.55	1.00-9.00
Strategies or steps for SBE (SoS)	499	5.05	0.72	2.00-7.00
Barriers to SBE (BtE)	500	11.35	2.97	.00-14.00
Attitudes toward SBE (ASBE)	500	16.59	3.69	.00-24.00
Attitudes of health care personnel about breast cancer (AHCPBC)	500	3.31	1.07	.00-4.00

Six out of nine measures KABP were found to have higher average values among women. These include overall knowledge about breast cancer (mean=14.32; SD=2.11), purpose or reasons for self breast examination (mean=13.08; SD=1.54); characteristics of women highly recommended for SBE (mean=6.96; SD=1.55); strategies or steps for SBE (mean=5.05; SD=0.72); barriers to SBE (mean=11.35; SD=2.97); and attitudes of health care personnel about breast cancer (mean=3.31; SD=1.07). We further analyzed all nine KABP indexes according to residence location and age groups (18-39 vs. 40+).

Table 3 provides the mean differentials of all KABP measures by location of residence and the respondents' age groups. The mean values of all KABP measures were found to be significantly different between rural and urban women except for the attitudes toward self breast examination. For example, urban women tend to have considerably higher overall knowledge about breast cancer than the overall knowledge of women living in rural areas. This is also true regarding the frequency and timing of SBE. However, other measures such as KCS, PoRs, CoW, SoS, BtE, and SHCPBC tend to be higher among rural women than those living in urban areas. 

**Table 3:** Mean of indicators by age and location SBE - self breast examination

Indicators	Rural	Urban	p-value	18-39	40+	p-value
Knowledge about breast cancer (KBC)	13.9	14.75	<0.01	14.14	14.56	<0.05
Knowledge about breast cancer screening (KCS)	2.99	2.83	<0.05	2.89	2.94	0.401
Frequency and timing (F&T)	20.34	23.97	<0.01	22.41	21.81	0.159
Purpose or reasons (PoRs)	13.52	12.64	<0.01	12.95	13.26	<0.05
Characteristics of women (CoW)	7.36	6.57	<0.01	6.76	7.24	<0.01
Strategies or steps of SBE (SoS)	5.22	4.89	<0.01	4.97	5.16	<0.01
Barriers to SBE (BtE)	12.25	10.46	<0.01	11.38	11.31	0.805
Attitudes toward SBE (ASBE)	16.71	16.48	0.476	16.64	16.54	0.775
Attitudes of health care personnel about breast cancer (AHCPBC)	3.43	3.18	<0.05	3.23	3.4	0.073

Mean differentials based on age group showed somewhat a different trend on these measures. Four out of nine measures found significant differences between women in the 18-39 age group and women in the 40+ age group. In particular, results indicated that women in the older age group (40+) reported higher mean knowledge about breast cancer than the overall knowledge among women in the 18-39 age group. Similar results are also found to be true for PoRs, CoW, and SoS. In other words, women in 40+ age groups tend to have an advantage on overall knowledge about breast cancer and specific aspects such as women's characteristics, purpose, reasons, strategies, and steps on SBE.

## Discussion

Given the increasing trends in breast cancer incidence and mortality and the high cost of screening and treatment, our study results highlight two broad but specific areas where federal and local agencies can implement basic prevention measures to break the ongoing trend. Our study results showed widespread differentials in basic knowledge and behaviors on self breast examination practices among women living in rural and urban locations in Nicaragua. For example, while knowledge about breast cancer regarding the timing and frequency of SBE were more common among urban women, barriers regarding SBE tend to be higher among rural women. Strategies incorporating locally trained women in the forefront of dissemination of the importance and significance of SBE and providing details on the timing and frequency of SBE can significantly impact the overall effort to curb breast cancer incidence in Nicaragua. Despite the family and community health care model, which implies home visits from the medical team in rural areas, most of these visits were related to detecting pregnancies, identifying children at risk, and some other chronic illness such as diabetes and hypertension. Healthcare knowledge varies significantly among individuals who live in rural and urban settings. In Nicaragua, the rural sectors are often located far from other healthcare resources [21,22]. This issue continues making it difficult for breast cancer prevention programs for those women living in these rural areas. Further emphasis must be taken into account in building prevention efforts targeting resource-poor settings in rural communities to improve the cancer screening practices among women in rural areas of Nicaragua. 

Another contribution of our study is targeting cancer prevention efforts to younger age groups of women to lower breast cancer incidence in the future. For instance, our study results indicated that while younger women reported significant and lower overall knowledge about breast cancer, purpose and reasons for SBE, characteristics of women who tend to be at higher risk for BC, and strategies and steps one take to perform SBE tended to be higher among 40+ age group compared to women who were in 18-39 age group. While clinical breast examination and mammograms are generally available at the public primary health care level and private care facilities, women’s understanding of accessing and utilizing such services must be initiated to address the age and location differentials in seeking such services. It is reported in previous studies that breast cancer treatment in Nicaragua, including surgery, chemotherapy, and all other recommended interventions, would cost approximately $13,000-$20,000 in a society where approximately 60 percent of the rural and 36.5 percent of the urban population were living in poverty in 2014 [17,22].

There are a few limitations to be mentioned in the study. First, given the cross-sectional nature and the data are collected from two locations of the country, one must be aware of the generalizability of the results to the rest of the country. Second, the present study did not include questions on ever conducting SBE or if women ever received mammograms. These questions would have further helped researchers and policymakers understand differentials in knowledge and behavioral practices among women regarding breast cancer prevention efforts.

## Conclusions

While the National Health Plan 2015-2021 of Nicaragua has recommended priorities for prevention services and strategies for improving the quality and accessibility of health care services, specific recommendations for breast cancer prevention were not included in the priority list. To further impact the incidence and mortality due to breast cancer, it is important to strengthen family and community health teams to educate the population during home visits and empower midwives and community leaders, emphasizing the importance of early detection and thus gradually mitigating cultural barriers around the disease. Our study results indicated that policies and programs targeting households in rural areas and to younger women in promoting the importance and significance of self-breast examination would make a significant and sustaining impact on nation’s effort to reduce the disparity, giving more relevance to SBE education to all women could warranty the identification of breast abnormalities at an earlier phase. This can be achieved by the health care staff at the Family and Community Health centers in Nicaragua during the women’s routine and follow-up visits as part of the health promotion activities.

## References

[1] O'Mahony M, Comber H, Fitzgerald T (2017). Interventions for raising breast cancer awareness in women. Cochrane Database Syst Rev.

[2] Woolston C (2015). Breast cancer. Nature.

[3] World Health Organization (2018). Global status report on alcohol and health. Health.

[4] Liu J, Wang J (2020). Disability-adjusted life-years (DALYs) for breast cancer and risk factors in 195 countries: findings from Global Burden of Disease Study 2017 [PREPRINT].

[5] Smith RA, Andrews K, Brooks D (2016). Cancer screening in the United States, 2016: a review of current American Cancer Society guidelines and current issues in cancer screening. CA Cancer J Clin.

[6] Becker S (2015). A historic and scientific review of breast cancer: the next global healthcare challenge. Int J Gynaecol Obstet.

[7] Angel-Urdinola D, Cortez R, Tanabe K (2008). Equity, access to health care services and expenditures on health in Nicaragua. http://hdl.handle.net/10986/13667.

[8] Di Sibio A, Abriata G, Forman D, Sierra MS (2016). Female breast cancer in Central and South America. Cancer Epidemiol.

[9] Kaiser B, Bouskill K (2013). What predicts breast cancer rates? Testing hypotheses of the demographic and nutrition transitions. J Popul Res.

[10] Luciani S, Cabanes A, Prieto-Lara E, Gawryszewski V (2013). Cervical and female breast cancers in the Americas: current situation and opportunities for action. Bull World Health Organ.

[11] Graves KD, Huerta E, Cullen J (2008). Perceived risk of breast cancer among Latinas attending community clinics: risk comprehension and relationship with mammography adherence. Cancer Causes Control.

[12] Leon-Rodriguez E, Molina-Calzada C, Rivera-Franco MM, Campos-Castro A (2017). Breast self-exam and patient interval associate with advanced breast cancer and treatment delay in Mexican women. Clin Transl Oncol.

[13] White RM, Camper L (2016). Breast self-examination education among Dominican women: a pilot study. Int J Health Promot Educ.

[14] Duda RB, Bhushan D (2011). Teaching rural women in Nicaragua the principles of breast health. J Cancer Educ.

[15] Doede AL, Mitchell EM, Wilson D, Panagides R, Oriá MO (2018). Knowledge, beliefs, and attitudes about breast cancer screening in Latin America and the Caribbean: an in-depth narrative review. J Glob Oncol.

[16] Tsu VD, Jeronimo J, Anderson BO (2013). Why the time is right to tackle breast and cervical cancer in low-resource settings. Bull World Health Organ.

[17] ECLAC ECLAC (2021). CEPALSTAT database and statistical publications. https://statistics.cepal.org/portal/cepalstat/index.html?lang=en.

[18] Betanco U (2009). Conocimientos, actitudes y prácticas sobre el autoexamen de mamas en mujeres de 20-54 años que acuden al centro de salud "María del Carmen Salmerón”. Chinandega. León: UNAN-León. Tesis (Doctor.

[19] Bonilla C (2007). Conocimientos, actitudes y prácticas sobre autoexamen de mama de las mujeres atendidas en el Servicio de Maternidad del Hospital Fernando Vélez Páiz. Conocimientos, actitudes y prácticas sobre autoexamen de mama de las mujeres atendidas en el Servicio de Maternidad del Hospital Fernando Vélez Páiz.

[20] López S, Carlos J, Peralta A, Ramón J (2006). Conocimientos, actitudes y prácticas sobre auto-examen de mama y mamografía como detección precoz del cáncer de mama en mujeres leonesas. http://riul.unanleon.edu.ni:8080/jspui/handle/123456789/2131.

[21] Steppe JD, Blake BJ, Dyal MA, Bailey TS, Porter KJ, Thompson J (2017). Conducting a health education needs assessment in rural Nicaragua. Health Education Journal.

[22] Foley O, Dizon D, Schmeler K, Del Carmen M (2015). Development of a breast cancer advocacy and access health program in Nicaragua. Breast J.

